# Oleanolic acid ameliorates high glucose-induced endothelial dysfunction via PPARδ activation

**DOI:** 10.1038/srep40237

**Published:** 2017-01-09

**Authors:** Zihui Zhang, Manli Jiang, Xinya Xie, Haixia Yang, Xinfeng Wang, Lei Xiao, Nanping Wang

**Affiliations:** 1 Cardiovascular Research Center, Xi’an Jiaotong University, Xi’an, 710061, China; 2 The Advanced Institute for Medical Sciences, Dalian Medical University, Dalian, 116044, China

## Abstract

Oleanolic acid (3β-hydroxyolean-12-en-28-oic acid, OA) is a pentacyclic triterpenes widely distributed in food, medicinal plants and nutritional supplements. OA exhibits various pharmacological properties, such as hepatoprotective and anti-tumor effects. In this study, we analyzed the effect of OA on endothelial dysfunction induced by high glucose in human vascular endothelial cells (ECs). Western blotting showed that OA attenuated high glucose-reduced nitric production oxide (NO) as well as Akt-Ser^473^ and eNOS-Ser^1177^ phosphorylation in cultured human umbilical vein ECs (HUVECs). Next, luciferase reporter assay showed that OA activated peroxisome proliferators-activated receptor δ (PPARδ) activity. Quantitative reverse transcriptase PCR (qRT-PCR) demonstrated that OA increased the expressions of PPARδ target genes (PDK4, ADRP and ANGPTL4) in ECs. Meanwhile, the induced expressions of PDK4, ADRP and ANGPTL4 by OA were inhibited by GSK0660, a specific antagonist of PPARδ. In addition, inhibition of PPARδ abolished OA-induced the Akt-Ser^473^ and eNOS-Ser^1177^ phosphorylation, and NO production. Finally, by using Multi Myograph System, we showed that OA prevented high glucose-impaired vasodilation. This protective effect on vasodilation was inhibited in aortic rings pretreated with GSK0660. Collectively, we demonstrated that OA improved high glucose-impaired endothelial function via a PPARδ-mediated mechanism and through eNOS/Akt/NO pathway.

Oleanolic acid (OA), a pentacyclic triterpenoid compound, present in many fruits and vegetables, such as olive leaves, grape, clove and pomegranate flowers^1^ exhibits a wide range of pharmacological and biochemical effects^2^,^3^. OA has received much attention, and is being marketed as therapeutic drug for the treatment of liver diseases, obesity associated insulin resistance, hypertension, atherosclerosis^4^,^5^. Especially, OA has been shown to possess promising anti-diabetic effects in various *in vitro* and *in vivo* models, as well as the ability to reduce blood pressure, blood glucose levels, total cholesterol, triglyceride, low density lipoprotein, and to increase the plasma insulin and high density lipoprotein levels^1^,^6^. However, the signal pathways underlying these effects remain to be elucidated.

Substantial clinical and experimental evidence suggest that both diabetes and insulin resistance cause endothelial dysfunction, which is considered the earliest predictive factor for diabetes^7^,^8^. One of the main targets against endothelial dysfunction is to improve endothelium-dependent vasodilatation. Nitric oxide (NO) is of critical importance as a mediator of vascular tone and blood pressure. Loss of NO bioavailability is a cardinal feature of endothelial dysfunction^9^,^10^. Several factors contribute to loss of NO bioavailability in endothelial dysfunction states, including both reduced NO synthesis and NO scavenging by reactive oxygen species (ROS)^11^. In ECs, NO is produced by endothelial nitric oxide synthase (eNOS), which catalyzes the oxidation of L-arginine to produce NO. The activity of eNOS can be regulated by a number of post-translational modifications. Among them, protein kinase B (Akt) induces eNOS-Ser^1177^ phosphorylation to modulate endothelial NO production in response to a wide variety of stimuli^12^,^13^.

PPARδ is a member of ligand-activated nuclear receptor transcription factors superfamily, which is ubiquitously expressed with high levels in placenta, skeletal muscles, and adipose tissue. PPARδ is also expressed in the vascular cells including ECs, smooth muscle cells and macrophages^14^. PPARδ plays important roles in various physiological vascular processes such as apoptosis, survival, angiogenesis and inflammation^15^. PPARδ also promotes vasodilatation by stimulating NO production^16^. Recently, we demonstrated an endothelial-protective effect of synthetic PPARδ agonists in diabetic mice through PI3K/Akt/eNOS signaling^17^. In this study, we sought to investigate the effects of a natural product OA on high glucose-impaired NO production and vasorelaxation.

## Results

### OA improved high glucose-induced NO reduction in BAECs

**Figure 1:**
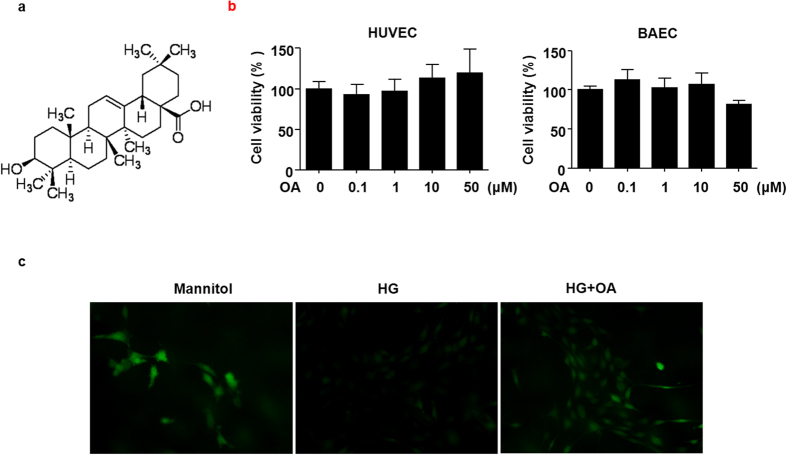
OA improved high glucose-induced NO reduction in BAECs. (**a**) The chemical structure of OA. (**b**) HUVECs and BAECs were treated with indicated concentrations of OA for 24 h, and cell viability was measured by MTT. All data were expressed as mean ± SEM of triplicate experiments. (**c**) BAECs were preincubated with or without OA (10 μM) for 12 h, then, treated with high glucose (HG, 30 mM, 12 h), mannitol served as vehicle control to HG. NO was detected by using DAF-FM diacetate (40 × objective).

Endothelial dysfunction is implicated in vascular complications of diabetic patients^18^. To study the effects of OA (Fig. 1a) on endothelial function in ECs, we evaluated the cytotoxicity of OA on HUVECs and BAECs firstly by using the MTT assay. Both HUVECs and BAECs were treated with the indicated concentrations (0.1–50 μM) of OA for 24 h. As shown in Fig. 1b, at a concentration up to 10 μM caused no decrease in cell viability in either cell types. Thus, this concentration was used in the following cell-based experiments. Then we examined the effect of OA on the endothelial production of NO using the NO-sensitive dye DAF-FM diacetate. As shown in Fig. 1c, treatment with high glucose (HG, 30 mM, 12 h) significantly reduced NO production compared with mannitol control. Pretreatment with OA (10 μM) effectively restored the NO production in BAECs.

### OA attenuated the high glucose-induced impairment of Akt-Ser^473^ and eNOS-Ser^1177^ phosphorylation

**Figure 2:**
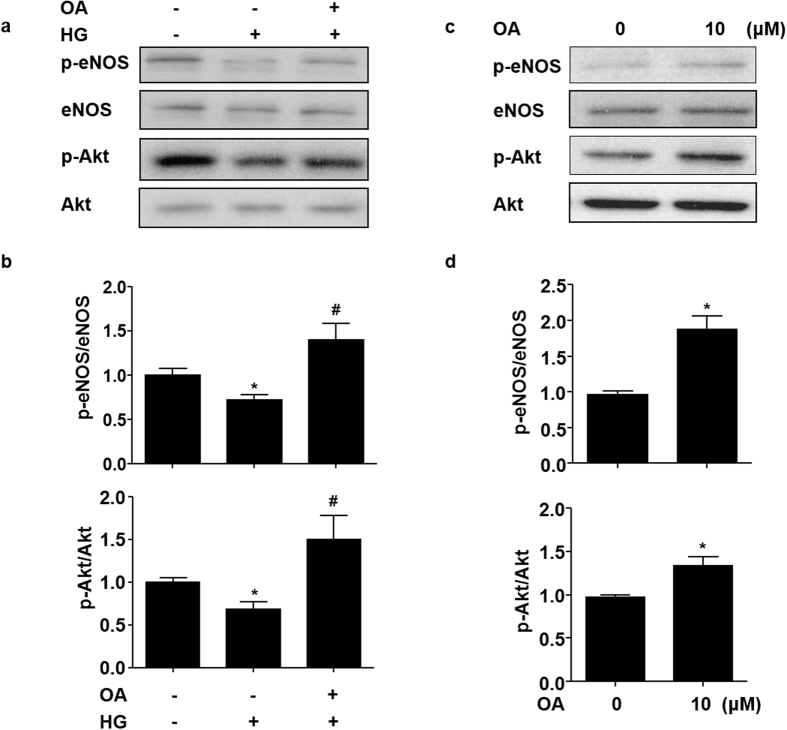
OA attenuated the high glucose-induced impairment of Akt-Ser^473^ and eNOS-Ser^1177^ phosphorylation. (**a**) HUVECs were pretreated with or without OA (10 μM) for 12 h, and then exposed to normal (5 mM) or high (30 mM) glucose for 12 h. Protein levels of p-eNOS, eNOS, p-Akt and Akt were detected by using western blotting. (**b**) Quantification of p-eNOS/eNOS and p-Akt/Akt. (**c**) HUVECs were stimulated with OA (10 μM) for 24 h, cell lysates were analyzed to determine p-eNOS, eNOS, p-Akt and Akt protein levels by using western blot. (**d**) Quantification of p-eNOS/eNOS and p-Akt/Akt levels in HUVECs. Data were shown as mean ± SEM of independent experiments. *P < 0.05 *vs.* vehicle control. ^#^P < 0.05 *vs.* HG.

Phosphorylation of Akt at Ser^473^ plays a critical role in the transduction of insulin signaling and, in turn, phosphorylates its downstream substrate eNOS at Ser^1177^ to increase eNOS activity^13^,^19^. In HUVECs, HG treatment suppressed the phosphorylation of both Akt-Ser^473^ and eNOS-Ser^1177^. However, OA attenuated the suppression of Akt and eNOS phosphorylation by HG (Fig. 2a and b). As shown in Fig. 2c and d, OA could increase the basal levels of Akt-Ser^473^ and eNOS-Ser^1177^ phosphorylation.

### OA activated PPARδ in ECs

**Figure 3:**
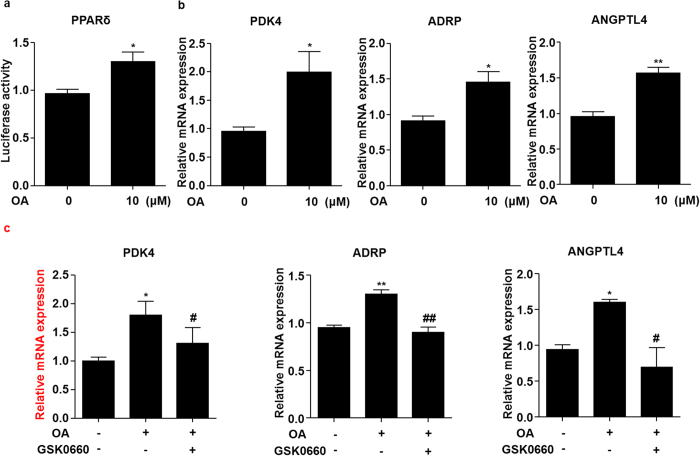
OA activated PPARδ in ECs. **(a**) BAECs were transfected with PPRE-luc and PPARδ plasmids and then treated with OA (10 μM) for 24 h. The luciferase activities were shown as fold changes in relation to the control. (**b**) HUVECs were stimulated with OA (10 μM) for 24 h, PDK4, ADRP and ANGPTL4 mRNA levels were assessed by using qRT-PCR. **(c)** HUVECs were pretreated with GSK0660 (1 μM) for 1 h, then exposed to OA (10 μM) for 24 h, mRNA levels of PDK4, ADRP and ANGPTL4 were assessed. *P < 0.05, **P < 0.01 *vs.* vehicle control. ^#^P < 0.05, ^##^P < 0.01 *vs.* OA.

We previously demonstrated a protective effect of PPARδ on endothelial function in diabetic mice through activating the Akt/eNOS signaling pathway^17^. Thus, we examined whether OA activated PPARδ in ECs. BAECs were transfected with the PPRE-driven luciferase reporter and PPARδ expression plasmid before exposed to OA. The reporter assay showed that OA increased the luciferase activity of PPARδ (Fig. 3a). Further, we examined the effects of OA on the expressions of the endogenous PPARδ target genes including pyruvate dehydrogenase kinase 4 (PDK4), adipose differentiation-related protein (ADRP) and angiopoietin-like protein 4 (ANGPTL4). As shown in Fig. 3b, the mRNA expressions of PDK4, ADRP and ANGPTL4, were increased by OA. In addition, pretreatment with GSK0660, a selective antagonist of PPARδ, effectively abrogated the induction of PDK4, ADRP and ANGPTL4 by OA (Fig. 3c), suggesting a PPARδ-specific mechanism.

### OA attenuated the high glucose-induced impairment of NO production *via* PPARδ

**Figure 4:**
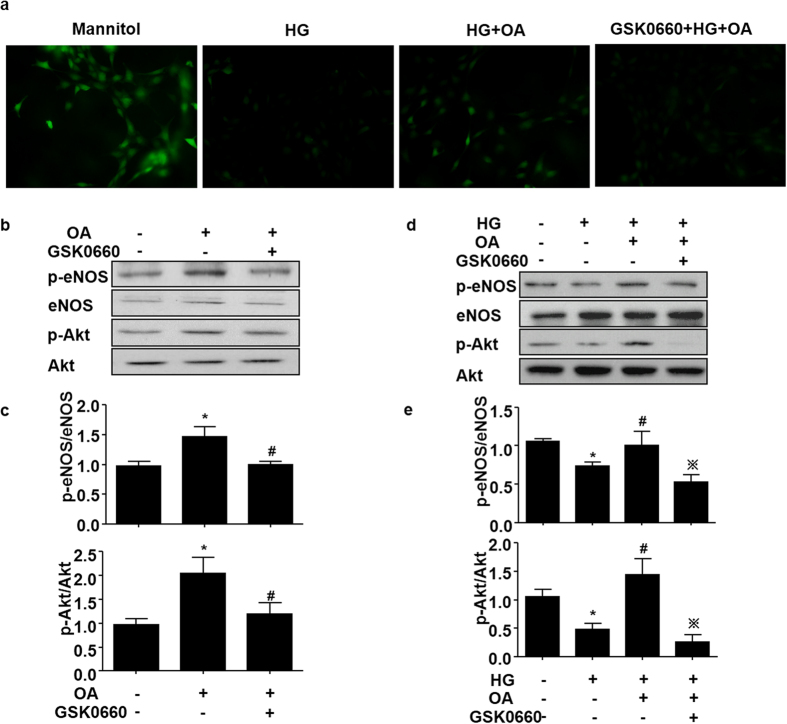
OA attenuated the high glucose-induced impairment of NO production *via* PPARδ. (**a**) BAECs were pretreated with GSK0660 (1 μM) for 1 h, and then stimulated with OA (10 μM) for 12 h by following normal (5 mM) or high (30 mM) glucose medium for 12 h. NO was detected by using DAF-FM probe (40 × objective). (**b**) HUVECs were treated as in (**a**). Protein levels of p-eNOS, eNOS, p-Akt and Akt levels were assessed. **(c)** Quantification of p-eNOS/eNOS and p-Akt/Akt levels. *P < 0.05 *vs.* vehicle control, ^#^P < 0.05 *vs.* OA. (**d**) HUVECs were pretreated with GSK0660 (1 μM) for 1 h, then exposed to OA (10 μM) for 24 h, protein levels of p-eNOS, eNOS, p-Akt and Akt levels were assessed. (**e**) Quantification of p-eNOS/eNOS and p-Akt/Akt levels. *P < 0.05 *vs.* vehicle control, ^#^P < 0.05 vs HG and ^※^P < 0.05 *vs.* OA.

In order to study whether PPARδ activity is required for the protective effect of OA on NO production against high glucose, HUVECs were pretreated with a selective PPARδ antagonist GSK0660 and then with OA (10 μM, 12 h) before the exposure to high glucose. The results demonstrated that, in the presence of GSK0660, OA failed to restore the high glucose impaired NO production (Fig. 4a). We further examined the role of PPARδ in the Akt and eNOS phosphorylation induced by OA. As shown in Fig. 4b and c, inhibition of PPARδ activity by GSK0660 significantly diminished the OA-induced eNOS-Ser^1177^ and Akt-Ser^473^ phosphorylation. In the presence of GSK0660, OA also failed to protect ECs against the high glucose-impaired phosphorylations of Akt and eNOS (Fig. 4d and e).

### OA enhanced endothelium-dependent vasodilatation via PPARδ

**Figure 5:**
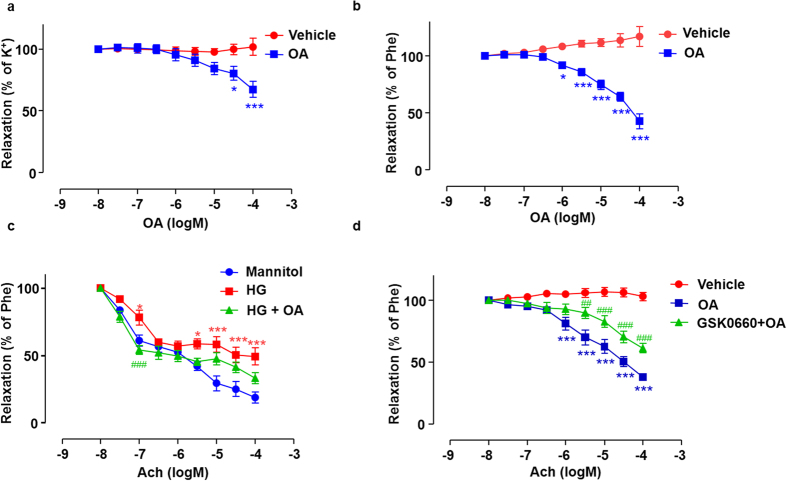
OA enhanced endothelium-dependent vasodilatation via PPARδ. Rat aortic arteries were pre-contracted by 60 mM K^+^ (**a**) or 10 μM Phe (**b**) followed by the cumulative addition of vehicle control or OA. Data were shown as mean ± SEM (n = 3. *P < 0.05, ***P < 0.001 *vs.* vehicle control). (**c**) Arterial rings were incubated in medium contained 30 mM glucose in the presence of 10 μM OA for 12 h, and ACh-induced vasodilatation was tested by using Multi Myograph System. Data are shown as mean ± SEM (n = 7). *P < 0.05, ***P < 0.001 *vs.* vehicle control, ^###^P < 0.05 *vs.* HG. (**d**) Arterial rings were incubated with GSK0660 (1 μM) for 1 h followed by the cumulative addition of OA pre-contracted by Phe (n = 5). ***P < 0.001 *vs.* vehicle control, ^##^P < 0.01, ^###^P < 0.001 *vs.* OA.

Compared with the time-matched vehicle control, OA elicited concentration-dependent relaxations pre-contracted by KCl or phenylephrine in the rat arterial segments (Fig. 5a and b). Next, aortic rings were exposed to high glucose co-incubated with or without OA for 12 h. Acetylcholine-induced endothelium-dependent vasorelaxation was impaired by high glucose (Fig. 5c). HG impaired blood vessel relaxation was attenuated by OA. However, the effect of OA was abolished when the arteries were pre-incubation with GSK0660 (Fig. 5d). These results suggest that OA improves endothelial relaxation and attenuates the high glucose impairment *via* the PPARδ activation.

## Discussion

In this study, we have demonstrated for the first time that OA, as a natural product, can ameliorate the high glucose-triggered endothelial function by activating the nuclear receptor PPARδ. Previous studies showed that pentacyclic triterpenes from fruits and plants could increase estrogen receptor activities^20^ and synthetic triterpenes has been identified as ligands for the PPARγ^21^. In addition, reporter gene assays showed that oleanolic acid increased PPARα activity in spontaneously transformed keratinocyte cell line HaCaT and CV-1 cells^22^. *Punica granatum* flower (PGF) and its component oleanolic acid enhanced the PPARα reporter activity in human embryonic kidney 293 cells, and this effect was completely suppressed by a selective PPARα antagonist^23^,^24^. We and others have previously demonstrated that synthetic PPARδ agonists such as GW501516 and GW0742 could ameliorate a number of pathological features associated with diabetes and metabolic syndrome. In ECs, these agonists inhibited the expression of pro-inflammatory genes^14^. In mouse models for atherosclerosis, synthetic agonists of PPARδ reduced atherosclerotic lesions^25^. In obese diabetic mice, the PPARδ agonists prevented fatty liver by repressing sterol regulatory element binding protein-1 (SREBP1) and improved vascular function^17^,^26^. These results clearly indicate that PPARδ is a promising target for treating metabolic syndrome and related cardiovascular diseases. However, clinical uses of such potent synthetic agonists have been seriously hindered largely due to the claimed cardiovascular adverse effects of PPARγ^27^. Therefore, searching natural compounds with PPARδ modulating properties and, in particular, vascular protective activity, represent a promising approach for metabolic syndrome and related vascular disorder, also in view of their advantageous effects in endothelial system and obesity prevention^28^.

Emerging evidence revealed the beneficial effects of OA on endothelial function in vascular disorders associated to metabolic diseases. OA induced vasodilatation in isolated aortas from both normotensive and spontaneously hypertensive rats^5^,^29^. NO was involved in the vasodilator responses of OA, and in ECs, OA induced phosphorylation of eNOS-Ser^1177^ to increase NO production^30^. Further studies have shown that OA increased the phosphorylation of Akt-Ser^473^ and led to eNOS activation^31^. These observations were largely consistent with our results. However, the mechanisms by which OA regulates Akt/eNOS pathway were poorly understood. Herein, we demonstrated that OA exerts the endothelial protective effects mainly by PPARδ activation. This notion was supported by its capacity of activating the PPAR-reporter (Fig. 3a) and the induction of multiple endogenous PPARδ target genes, PDK4, ANGPL4 and ADRP (Fig. 3b). More importantly, the effects of OA were attenuated by a selective PPARδ antagonist GSK0660 (Fig. 3c). It remains to be examined whether this pentacyclic triterpenoid serves as a *bona fide* ligand for PPARδ or enhances the transcriptional activity of PPARδ by a post-translational modification.

It is well known that exposure to high glucose impairs endothelial functions and decreases the NO production. These deleterious effects are attributed to the inhibition of expression and/or activity of eNOS^32^,^33^. PPARδ agonists could ameliorate the endothelium-dependent vasodilatation impaired by HG via activating PI3K/Akt/eNOS pathway in MAECs^17^. In the present study, we found that OA protected endothelial cells from high glucose injury (Fig. 1). This effect could also be inhibited by blocking PPARδ (Fig. 2). In endothelium-specific PPARδ deficient mice, both endothelium-dependent relaxations to ACh and endothelium independent relaxations to the NO donor were significantly impaired in the arteries, accompanied by decreased eNOS-Ser^1177^ phosphorylation^34^. It is suggested that OA restores HG-impaired endothelial function by a PPARδ-mediated activation of Akt/eNOS signaling pathways. It is worth noting that OA and PPARδ both possess an anti-oxidative property^35^. Under oxidative stress, eNOS undergoes uncoupling and produces superoxide rather than NO. GTP cyclohydrolase I (GTPCH I), a rate-limiting enzyme responsible for tetrahydrobiopterin (BH4) *de novo* synthesis, switches eNOS from uncoupled to coupled state to balance the NO and reactive oxygen species (ROS) generations^36^. GTPCH I protein level was up-regulated by GW501516, as well as the production of BH4 in endothelial progenitor cells^37^. Thus, the effects of OA on GTPCH I expression and eNOS coupling remain to be investigated.

In summary, this study provided evidence that OA, a component of pentacyclic triterpenes, is a natural modulator of PPARδ and ameliorates the high glucose impaired endothelial dysfunction. It is suggested that OA has a potential application in the treatment of diabetes related vascular diseases.

## Materials and Methods

### Ethics statement

The animal experiment was approved by the institutional review board of Xi’an Jiaotong University and performed in accordance with the institutional and national guidelines for the care and use of animals.

### Reagents

OA (93.4% in purity) was from ChromaDex (Irvine, CA, USA). The antibodies against phosphorylation eNOS (Ser^1177^), eNOS, phosphorylation Akt (Ser^473^), Akt were obtained from Cell Signaling Technology (Danvers, MA, USA). Antibodies against β-actin and horseradish peroxidase (HRP)-conjugated secondary antibody were from Santa Cruz Biotechnology (Santa Cruz, CA, USA). Fetal bovine serum (FBS) was from HyClone (Longan, UT, USA). Thiazolyl blue tetrazolium bromide (MTT) was from Sigma-Aldrich (St. Louis., MO, USA). 4-amino-5-methylamino-2′, 7′-difluorofluorescein (DAF-FM) diacetate was from Life Science Ltd. (Oregon, CA, USA). Polyvinylidene difluoride (PVDF) membranes for western blot analysis was purchased from Roche Diagnostics (Mannheim, Germany).

### Cell culture

Human umbilical vein endothelial cells (HUVECs) were cultured in medium 199 containing heparin (0.1 mg/ml), acidic fibroblast growth factor (FGF) (10 ng/ml), L-glutamine (2 mM), penicillin (100 U/ml), streptomycin (100 U/ml) and 20% FBS. Bovine aortic endothelial cells (BAECs) were maintained in Dulbecco’s modified Eagle medium (DMEM) with 10% FBS, penicillin (100 U/ml), streptomycin (100 U/ml).

### MTT Assay for Cell Viability

Cell viability was evaluated by MTT assay. HUVECs and BAECs were seeded in 96-well plates and cultured until 80% confluence. Then the cells were treated with the indicated concentrations of OA for 24 h before incubation with 5 mg/ml MTT at 37 °C in 5% CO_2_ atmosphere for 4 h. Next, the culture medium was removed and the formazan formed in the reaction was dissolved in 150 μl DMSO. Cell viability was presented as a percentage of the vehicle.

### Quantitative Reverse Transcriptase-PCR (qRT-PCR)

Total RNA was isolated using TRIzol (Invitrogen, Carlsbad, CA), converted to cDNA by iScript cDNA synthesis kit (Bio-rad, Hercules, CA). Real-time PCR was performed by using SYBR Green Supermixes (Bio-rad) and a 7500 Real-time PCR machine (Applied Biosystems, Foster City, CA). The reaction conditions consisted of: stage 1, 95 °C for 10 min; stage 2, 40 cycles of 95 °C for 30 s, 60 °C for 30 s and 72 °C for 30 s, which were concluded by the melting curve analysis process. Fold changes of gene expression were calculated using the 2^−ΔΔCt^ method. The primer sequences are listed in Table 1. Glyceraldehyde-3-phosphate dehydrogenase (GAPDH) was used as an internal control.

### Western blotting

Cellular proteins were extracted with lysis buffer (50 mM Tris-HCl, pH 7.5, 15 mM EGTA, 100 mM NaCl, 0.1% Triton X-100 and the protease inhibitors). Protein samples were separated on 10% SDS-PAGE gels, transferred to PVDF membranes, and incubated overnight at 4 °C with primary antibodies directed against phosphorylation eNOS (Ser^1177^) (1:1000), eNOS (1:1000), phosphorylation Akt (Ser^473^) (1:2000), Akt (Ser^473^) (1:2000) or β-actin (1:5000). After washing blots to remove excessive primary antibody binding, the membranes were incubated for 1 h with horseradish peroxidase (HRP)-conjugated secondary antibody (1:3000) at room temperature, then visualized by the ECL chemoluminescence system.

### Plasmids, Transfection and Reporter Assay

The plasmids expressing PPARδ and PPRE-TK-luciferase reporter contains 3 copies of PPAR-response elements (PPRE) from the acyl-coenzyme A (CoA) oxidase gene were transfected into subconfluent BAECs together with pRSV-gal, a plasmid expressing β-galactosidase by using Lipofectamine 2000 (Invitrogen, Carlsbad, CA, USA). 24 hours later, the cells were treated with or without OA. Cell lysates were harvested to measure the luciferase and β-gal activities.

### Detection of NO

NO release was measured with using DAF-FM diacetate. Briefly, BAECs seeded on glass coverslips and treated with different stimuli. By the end of treatment, the cells were incubated with DMEM containing DAF-FM (5 μM) for 30 min in the dark at 37 °C and then washed with PBS. Images were obtained using fluorescence microscopy (Olympus America Inc., NY, USA).

### Vascular reactivity

Aortic rings (3 mm) were dissected from Sprague-Dawley rats and incubated with different treatments at 37 °C in 5% CO_2_, then mounted in Multi Myograph System (Danish Myo Technology A/S, Denmark) to measure the isometric force. During the whole experiment, the solution was continuously oxygenated with a gas mixture of 95% O_2_ plus 5% CO_2_. To determine the vasodilatory effect of OA, KCl (60 mM) or phenylephrine (Phe, 10 μM) was used to constrict arterial rings in advance. After sustained contraction was obtained, the concentration-dependent responses of OA (0.01–100 μM) were examined. In order to evaluate the role of high glucose, arterial rings were incubated in medium containing 30 mM glucose in the presence or absence of 10 μM OA to examine the acetylcholine-induced vasodilation.

### Statistical analysis

Quantitative data are expressed as mean ± SEM. Student’s *t* test and ANOVA were used to analyze the statistical significance for the differences between two or among more groups, respectively. The dose response curves were analyzed by using two-way ANOVA followed by Bonferroni post-tests. P < 0.05 was considered significant. Non-quantitative results were representative of at least three independent experiments.

## Additional Information

**How to cite this article**: Zhang, Z. *et al*. Oleanolic acid ameliorates high glucose-induced endothelial dysfunction via PPARδ activation. *Sci. Rep.* **7**, 40237; doi: 10.1038/srep40237 (2017).

**Publisher's note:** Springer Nature remains neutral with regard to jurisdictional claims in published maps and institutional affiliations.

## Figures and Tables

**Table 1:** List of primer pairs used for qRT-PCR.

Primer	Forward (5′-3′)	Reverse (5′-3′)
ADRP	TCAGCTCCATTCTACTGTTCACC	CCTGAATTTTCTGATTGGCACT
ANGPTL4	AAAGAGGCTGCCCGAGAT	GCGCCTCTGAATTACTGTCC
PDK4	AGGTCGAGCTGTTCTCCCGCT	GCGGTCAGGCAGGATGTCAAT
GAPDH	ACCACAGTCCATGCCATCAC	TCCACCACCCTGTTGCTGTA
